# Executive Resources and Item-Context Binding: Exploring the Influence of Concurrent Inhibition, Updating, and Shifting Tasks on Context Memory

**DOI:** 10.5709/acp-0176-9

**Published:** 2015-09-30

**Authors:** Marek Nieznański, Michał Obidziński, Emilia Zyskowska, Daria Niedziałkowska

**Affiliations:** Institute of Psychology, Cardinal Stefan Wyszyński University in Warsaw, Poland

**Keywords:** context memory, executive resources, inhibition, updating, shifting

## Abstract

Previous research has demonstrated that context memory performance decreases as a result of cognitive load. However, the role of specific executive resources availability has not been specified yet. In a dual-task experiment, participants performed three kinds of concurrent task engaging: inhibition, updating, or shifting operations. In comparison with a no-load single-task condition, a significant decrease in item and context memory was observed, regardless of the kind of executive task. When executive load conditions were compared with non-specific cognitive load conditions, a significant interference effect was observed in the case of the inhibition task. The inhibition process appears to be an aspect of executive control, which relies on the same resource as item-context binding does, especially when binding refers to associations retrieved from long-term memory.

## Introduction

Episodic memories include at least two classes of information: features that are central to the observer, and details of associated context. Accurate performance in context memory tests seems to require not only memory for particular contextual features but also depends on cognitive processes that bind these details with item information (cf. Chalfonte & Johnson, 1996). In this study, we expected that successful binding of central and contextual information requires executive resources of working memory (WM, cf. Mammarella & Fairfield, 2008). The concept of WM is understood here according to the classical model by Baddeley and Hitch (1974), as a multicomponent system, the function of which is not restricted to temporary storage but also refers to manipulation of information. The processing component, the central executive, is aided by two subsidiary slave systems, one holding verbal and acoustic information, and another holding visuospatial information. Baddeley (2000), proposed an additional storage system, called the episodic buffer, which has binding as one of its main functions (see Allen, Baddeley, & Hitch, 2006). It may be assumed that during the encoding phase of a memory experiment, binding of context information and item information occurs in the episodic buffer. According to Baddeley, the central executive of WM can influence the content of the episodic store by directing attention to a given source of information, including information retrieved from long-term memory (LTM). Therefore, it seems that executive processes are involved in item-context integration that occurs in the episodic buffer. A disturbance of executive processes induced by the concurrent task may impair binding of information in the episodic buffer. For example, executive control is required to inhibit inappropriate associations between item and context information that may be retrieved from LTM. Although we focus here on Baddeley’s model of WM, other approaches may also be useful as a theoretical background. For example, in Cowan’s (1999) embedded-process model of WM, executive processes control the focus of attention. Engaging these processes in a concurrent task should impair entering information into the focus of attention, and consequently disturb the formation of a composite trace. In a similar framework by Oberauer (2009), one of the key executive functions is to adjust a threshold which regulates projection of representations activated in LTM into the central components of WM. Performing a concurrent executive task may disturb this regulation, inhibiting retrieval of the associations between item and context information that are stored in LTM. Suggestions concerning the close relationship between context memory deficits and executive dysfunctions are also supported by clinical neuropsychology literature (for a review see El Haj & Allain, 2012), however, what we seek in our research is experimental rather than clinical data.

In the first place, it seems necessary to clearly define that by cognitive load we mean conditions that demand controlled processing. Performance under cognitive load depends on the capabilities of the central executive (in terms of the Baddeley & Hitch, 1974, theory) or controlled attention (as explained by Engle, Kane, & Tuholski, 1999). When two tasks have to be executed simultaneously or alternately, they interfere with each other competing for general and/or specific attentional resources. In previous studies, cognitive load was manipulated in two different ways: The first manipulation involved a generation operation, the second used a concurrent distracter task. Many studies have shown that generating a word during encoding, in comparison with reading it, results in worse memory for its intrinsic context (e.g., font colour) (e.g., Mulligan, 2004, 2011; Mulligan, Lozito, & Rosner, 2006; Nieznaski, 2013, Experiment 1). Moreover, the more difficult the generation task that was used while encoding, the worse was the context-memory performance (Nieznaski, 2011, 2012). Recently, in a dual-task experiment, Nieznaski (2013, Experiment 2) has shown that dividing attention during encoding results in a lower context memory in comparison with a full attention condition. In this experiment, during the study phase of a memory task, participants performed the random number generation (RNG) task as a concurrent task. A decrease in context memory due to the cognitive load was observed, and it was more salient when item-context binding was difficult than when it was easy. More specifically, memory for font colour was poorer for words whose meanings were pre-experimentally unrelated to their font colours (e.g., the word grass printed in red font) than for words whose meanings were related to their colours (e.g., the word heart printed in red font). In general, previous research has shown that cognitive (attentional) resources are required at encoding in order to bind item and context information. The RNG task used in the study mentioned above is a heterogeneous task that involves diverse executive processes (e.g., Brown, Collier, & Night, 2013; Wierzcho, Gaillard, Asanowicz, & Cleeremans, 2012). Therefore, this task only suggests involvement of executive processes, without specifying which one is responsible for the interference with binding. The purpose of the current study is to tentatively explore what types of cognitive resources are required for successful binding. In a well-known analysis, Miyake, Friedman, Emerson, Witzki, and Howerter (2000) indicated that three partially separable factors (i.e., inhibition of the prepotent response, mental set shifting, and information updating) support executive functions. Confirmatory factor analysis showed that a three-factor model of executive functions fits the data significantly better than a one-factor or a two-factor model (see also e.g., Was, 2007). This three-component approach is also used in the current study. *Inhibition* is the ability to inhibit the automatic or dominant reactions to a presented stimulus when this is necessary for effective performance. This executive function is not connected with the inhibition of spreading activation (the reactive inhibition) but is an intended process of control over the prepotent reaction. *Shifting* is responsible for the ability to effectively switch between multiple tasks or mental states. Shifting is activated when a cognitive task forces us to change from one operation to another. In the process of switching between tasks, it is necessary to overcome proactive interference and negative priming. *Updating* is connected with monitoring and encoding of incoming information. However, it is not a passive storing but active manipulation of relevant information.

In the present study, the contribution of specific executive processing resources to the binding of context with item information was assessed using three different concurrent tasks. The experiment combined a context memory task, with secondary tasks emphasizing inhibition, updating, or shifting. Performance on the context memory task was compared between experimental conditions involving concurrent executive tasks, control conditions involving non-specific concurrent tasks, and a single-task condition. The working hypothesis in the present experiment was that item-context binding during the encoding phase of a memory experiment relies on the same processing resources that support the performance of one or more of the executive tasks. Therefore, concurrent tasks involving specific executive processes should result in worse context memory than non-specific dual-task conditions. Moreover, apart from specific executive resources, the availability of general cognitive resources should influence context memory performance, at least in difficult trials, as suggested by research on negative generation effect in context memory (Nieznaski, 2013, Experiment 1).

## Method

### Participants

One hundred and twenty-nine undergraduate students participated in the experiment in exchange for course credits. They were randomly assigned to four groups: one single-task control group (*N* = 21), and three dual-task groups (*N* = 36, each). All the participants were recruited from a population of second- and third-semester psychology students of Cardinal Stefan Wyszyński University in Warsaw. The great majority of participants were 20 years old and 75% of them were women.

### Materials and procedure

#### Stimuli

A set of 120 nouns was prepared for the experiment. Six lists of 20 words each were selected on the basis of rating lists of the most frequent associations to six colour names: blue, red, yellow, grey, green, and pink. These association lists were obtained from a group of 77 students, none of which took part in the present experiment. The students were asked to write down the strongest associations for each colour name. The colours were arranged in six different orders on sheets delivered to students, and each version was given to approximately an equal number of students. The responses for each colour were ranked from the most to the least frequent associations, the top 20 of which were selected as stimuli for the present study. Some frequent associations had to be replaced in case that they appeared as associations to more than one colour.

#### Procedure

Each participant took part in two consecutive sessions. In each session, three words served as buffers at the beginning and three at the end of the study list while 36 words were targets (associations to 3 colours × 12 words). During the study phase, at the first session, half of the words were presented in a red font and the other half in a blue font. At the second session, the font colours were grey and green. Three versions of slides were prepared and counterbalanced across participants so that each word on a list appeared in red and blue (the first session) (or grey and green during the second session) font colours or as a distracter equally often. The test list consisted of 54 words—that is, 36 target words were intermixed with 18 new words, all presented in black font. For each word, participants were asked to recognise whether it was presented in red or blue font (the first session), or grey or green (during the second session) at the time of the study, or whether it was a new word.

The study trials used in the experiment can be categorised into three classes: (a) words whose meaning was semantically related to their font colour (e.g., *heart* printed in red), (b) words whose meaning was unrelated to their font colour but related to another colour used during the study phase (e.g., *heart* printed in blue)—this class was labelled *opposite trials*, and (c) words whose meaning was related to a third colour that was not used during the study (e.g., *sun* is related to yellow but was printed in blue)—this class was labelled *neutral trials*. The two categories of unrelated trials (i.e., opposite and neutral) were differentiated in order to strengthen the reliability of the response-bias parameters’ estimation. However, it was expected that memory parameters do not differ between opposite and neutral trials.

Participants were divided into four groups: one single-task control group and three dual-task groups. In the single-task condition, participants solely performed two sessions of a memory task with no concurrent task. In the dual-task conditions, in one of two sessions, participants performed a task involving one of the executive processes (inhibition, updating, or shifting) concurrently with the study phase of a memory task. Moreover, participants from the three dual-task groups performed a control task concurrently with the study phase in one of two sessions—the task was similar in material and response type to the executive tasks used in the respective experimental dual-task conditions but was intended not to tap specific executive functions. Half of the participants performed the executive task as a concurrent task in the first session and the respective control task in the second session, the other half of the participants performed these tasks in the opposite order.

#### Concurrent tasks

In the single-task condition, participants were only told to read words and try to remember their font colours. The presentation time for each slide was 4.5 s. In the dual-task conditions, the executive tasks and their control counterparts were as follows:

(1) *Inhibition task*. In this task, participants were presented with arrays of one to three digits (e.g., 3 3), which were displayed on the slide just below the to-be-remembered word. Participants were asked to report (using a keyboard) the number of digits (i.e., 2) and ignore the identity of the digits (i.e., 3). The participant’s response appeared in the upper-left corner of the slide. Each slide was presented for 4.5 s. In the experimental session, the numerical information in all trials was incongruent—that is, the identity of the digits was different from the number of digits in the array (e.g., 2 2 2). In the control session, all the trials were congruent (e.g., 3 3 3). Therefore, there was no conflict between representations activated in memory by both aspects of the displayed stimuli in this condition.^1^ The Stroop-like interference effects in the number domain have been found in many studies. It seems that the numerical value is activated automatically. Therefore, it has to be inhibited in incongruent trials in order to produce a correct response (e.g., Flowers, Warner, & Polansky, 1979; Morton, 1969; Pavese & Umiltŕ, 1999; Windes, 1968).

(2) *Updating task*. Diverse methods have been recommended in the literature to study updating. Many of them include responding to a continuous series of items only after a fixed number of items has been presented (Brown et al., 2013). In the current experiment, we used a variation of this approach which is suitable for a concurrent task (cf. Fernandes & Moscovitch, 2000). Single digits were displayed on the slide just below the to-be-remembered word. The digits ranged from 1 to 8; even digits were displayed two times more frequently than odd digits. Each slide was presented for 4.5 s. In the experimental session, participants were asked to attend to whether the digit was odd or even, and to press a specific key on the keyboard whenever three or more consecutive digits were even. Thus, participants had to remember the three last digits and to update this sequence with each new digit presented on the slides. In the control session, the same stimuli were used, however, this time participants were asked to press a specific key whenever an odd digit was displayed on the slide. Thus, participants did not have to update their memory content. They only responded to the item currently presented on the screen.

(3) The *shifting task* was adapted from Jersild (1927, cited after e.g., Allport, Styles, & Hsieh, 1994; Piotrowski, Stettner, Wierzcho, Balas, & Bielecki, 2009). In this task, two pairs of digits were displayed on the slide just below the to-be-remembered word. A plus or minus sign was placed between the digits and each pair of digits was put before an equals sign and a question mark. Each slide was presented for 6.5 s. In the experimental dual-task session, one pair of digits had to be added and the other pair had to be subtracted (e.g., 3 + 4 = ?, 5 - 2 = ?). Thus, the participants had to shift between arithmetic operations. In the control dual-task session, a single arithmetic operation had to be performed during the whole session-that is, half of the participants only added digits (e.g., 3 + 4 = ?, 5 + 1 = ?), and the other half only subtracted digits (e.g., 4 - 3 = ?, 5 - 2 = ?) on each slide. The digits ranged from 1 to 9 and the outcome of arithmetic operations also ranged from 1 to 9. Participants were asked to indicate the outcomes of each operation using a keyboard. Their responses appeared in the upper-middle side of the slide.

#### Design

The independent variables in our experiment were encoding conditions (executive dual task vs. control dual task vs. single task) and the trial type of the word-context memory task (related vs. opposite vs. neutral). The trial type was manipulated within participants (and within lists). The kind of concurrent task (executive dual task vs. control dual task) was also manipulated within participants (but between sessions), and the absence versus presence of a concurrent task (executive dual task vs. single task) was manipulated between participants. The dependent variables were the parameters of the multinomial model measuring item detection, context memory, and response biases.

#### Data analysis

The data obtained in the memory task were analysed using the multinomial processing tree model, a method allowing for separate measurement of item and context memory as well as guessing biases. This is of special importance because some studies have suggested that better task performance for item-context related trials may be due to a decision bias rather than the result of better context memory. For example, Bayen, Nakamura, Dupuis, and Yang (2000) in one of their experiments used two pictures of faces (named Tom and Jim) as sources (contexts) presenting sentence statements. These statements were consistent with what a doctor might say, consistent with what a lawyer might say, and neutral with regard to either profession. The results showed that participants biased their decisions by relying on profession schemas. For example, when they did not remember who said “Are you taking any other medicine?”, they attributed this sentence to the person indicated as a doctor just before the test. Multinomial model analyses conducted by Bayen et al. (2000) provided evidence that correct source attributions for schema-consistent statements were due to guessing and not better context-memory.

**Figure B1.**
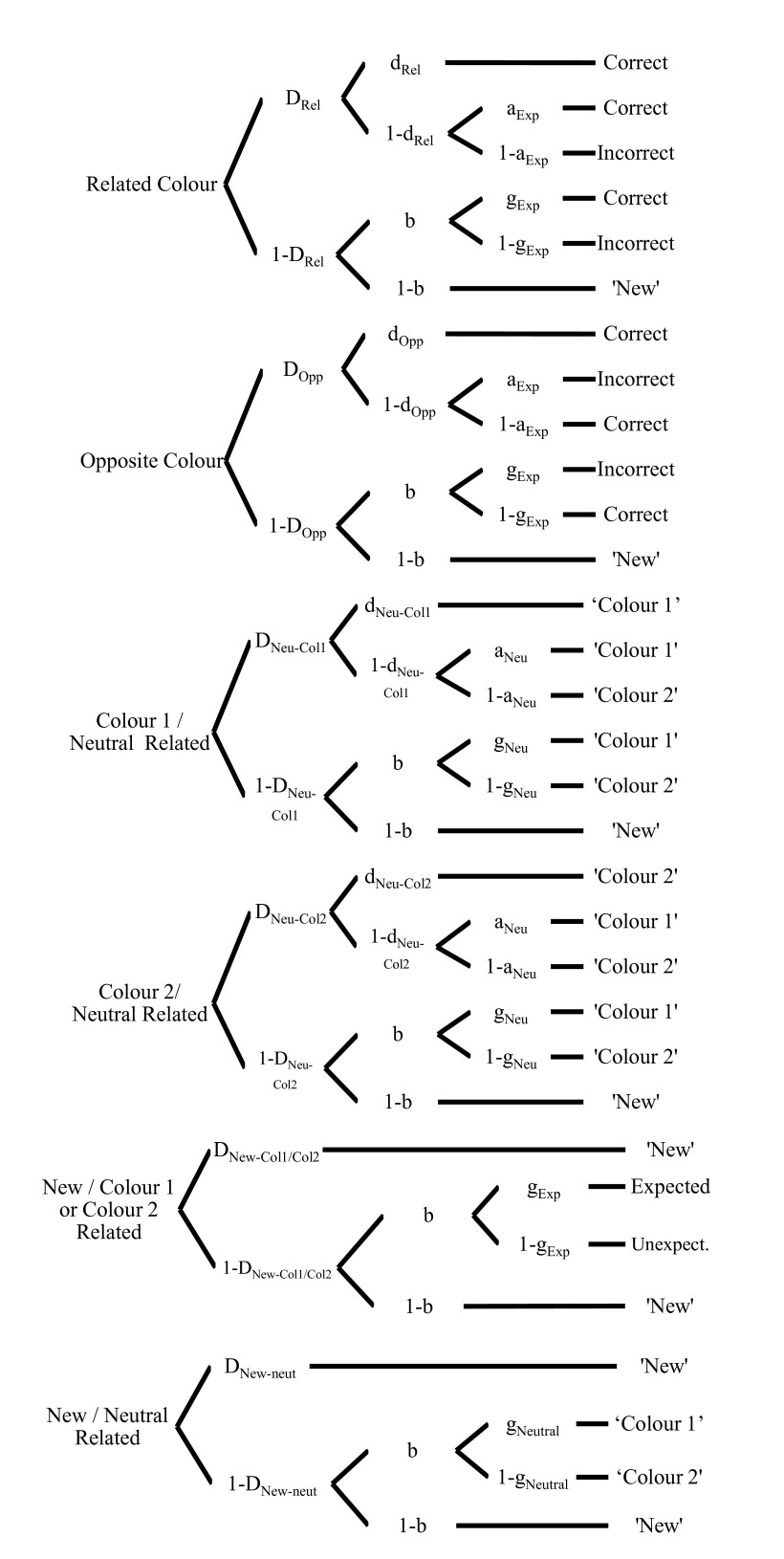
Processing tree multinomial model constructed for experiments with related, opposite and neutral trials (Nieznański, 2013). Item types are defined on the left, response types on the right side of the graph. Latent cognitive processes postulated by the model are the following: *D*_Rel_ = the probability of detecting an old item at related trials ; *D*_Opp_ = the probability of detecting an old item at opposite trials; *D*_Neu-Col1_ = the probability of detecting an old item related to the neutral colour but printed in Colour 1; *D*_Neu-Col2_= the probability of detecting an old item related to the neutral colour but printed in Colour 2; *D*_New-Col1/Col2_ = the probability of detecting new items related to Colour 1 or Colour 2; *D*_New-neut_ = the probability of detecting new items related to the neutral colour; *d*_Rel_ = the probability of correctly discriminating the context of an item at related trials; *d*_Opp_ = the probability of correctly discriminating the context of an item at opposite trials; *d*_Neu-Col1_ = the probability of correctly discriminating the context of an item related to neutral colour but printed in Colour 1; *d*_Neu-Col2_ = the probability of correctly discriminating the context of an item related to neutral colour but printed in Colour 2; *a*_Exp_ = the probability of guessing that a detected item was presented at study with an expected colour ; *g*_Exp_ = the probability of guessing that an undetected item was presented at study with an expected colour; *a*_Neu_ = the probability of guessing that a detected item related to neutral colour was presented in Colour 1; *g*_Neutral_ = the probability of guessing that an undetected item related to neutral colour was presented in Colour 1; *b* = the probability of guessing ‘old’ to undetected item.

A version of the multinomial model used in the present experiment was taken from Nieznaski (2013) that, in turn, was based on a two-high-threshold model of source monitoring developed by Bayen, Murnane, and Erdfelder (1996). In this model, latent cognitive processes of item detection, context memory, and three kinds of response biases are represented by separate parameters. The probabilities of correct detection of items from particular contexts are represented by parameter *D*. If an item was recognised as old, parameter *d* represents the probability of accurate context memory. The old items detected as old but not context-discriminated are subject to a guessing process; parameter *a* represents the probability of guessing that an item belongs to a particular context. If a new or old item is undetected, the observer may guess it is old with probability *b*. Then, *g* is the probability of guessing that this undetected item guessed to be an old one is from a particular context. In the version of the model used in the current experiment (see Figure B1 in Appendix B), each class of items has its specific detection and context memory parameters (e.g., *d*_Related_, *d*_Opposite_, *d*_Neutral_). Bias parameters are also specific to the class of tested items; for a word whose meaning is related to one of the study colours there may be a tendency to guess that it was printed in that colour at study (e.g., *a*_Expected_), whereas for a word whose meaning is related to a colour not used during the study, there should be no preference for one study colour over the other (*a*_Neutral_). The full version of the model contains too many parameters in relation to degrees of freedom in the data. Therefore, it is not mathematically identifiable and several restrictions had to be imposed on the parameters. These restrictions are described in the Results section of the experiment. The goodness of fit of the model to the empirical data was tested with the log-likelihood ratio statistic (*G*_2_) which is distributed asymptotically as a χ^2^ distribution. For more detailed information about multinomial modelling for context (source) memory tasks see, for example, Batchelder and Riefer (1990) or Bröder and Meiser (2007). An level of .05 was used for all statistical tests. At this level, *G*^2^(1) = 3.84 indicates a critical value. Response frequencies are shown in Appendix A. All computations were carried out with the *multiTree* computer program (Moshagen, 2010).

## Results

The mean percentages of correct responses in dual-task conditions are shown in Table 1. The participants were highly successful—their performance exceeded 90% correct responses in all dual-task conditions. On the one hand, the performance indicates their engagement in executing these tasks, on the other hand, it suggests the relative ease of these tasks.

Several restrictions were applied to the parameters of the model. First, it was assumed that item detection and context memory in opposite trials do not differ from item detection and context memory in neutral trials because, in both classes of trials, the word meaning is unrelated to its own colour (*D*_Opposite_ = *D*_Neutral_ = *D*_Unrelated_, and *d*_Opposite_ = *d*_Neutral_ = *d*_Unrelated_). Then, it was assumed that the probability of correct detection (*D*_Unrelated_) and context memory (*d*_Unrelated_) of words whose meaning is unrelated to the font colour does not differ depending on the colour that they were printed in during the study phase of the experiment. The next assumption common in source memory studies (see Bayen et al., 1996, p. 206) was that the distracter detection parameters for new words were equal to certain old-item detection parameters.^2^ Here, it was assumed that *D*_New/Neutral_ = *D*_Related_ and *D*_New/Related_ = *D*_Unrelated_. Alternatively, it may be assumed that distracter detection parameters are equal to some other old-item detection parameters. However, in comparison with alternative variants, the current version resulted in the best model fit. Moreover, restrictions were imposed on guessing parameters, wherein it was assumed that guessing tendencies are the same for undetected items and for detected but not context-discriminated items, *a* = *g*. Finally, the data sets obtained in the executive dual-task conditions and their respective control dual-task conditions were analysed using combined models. In such models, it was assumed that guessing biases do not depend on the kind of concurrent task (e.g., *b*_Inhibition_ task = b_Control_ to inhibition task). Such assumptions were confirmed by satisfactory model fits for most of the guessing parameter pairs, except the equality of *b* parameters in the shifting dual-task condition and its control condition, which, therefore, had to be estimated separately for each condition. All goodness of fit statistics were satisfactory after imposing the restrictions described above. Table 2 presents the log-likelihood ratio statistics obtained for multinomial models used in the experiment and the estimated parameter values.

**Table 1.** Percentages of Correct Responses in Concurrent Tasks

	Inhibition dual task	Control to Inhibition	Updating dual task	Control to Updating	Shifting dual task	Control to Shifting
Mean (*SD*)	98,61 (2,44)	99,54 (1,24)	95,83 (6,88)	99,46 (1,46)	93,42 (7,47)	91,65 (5,98)

*Note*. *SD* = standard deviation (values in parentheses).

**Table 2.** Parameter Estimates and *G*^2^ Goodness-of-Fit Values Obtained in the Context Memory Experiment

	Single-task condition	Dual-task conditions					
Concurrent task	Single task	Inhibition dual task	Control to Inhibition	Updating dual task	Control to Updating	Shifting dual task	Control to Shifting
Parameter	Estimate [*SE*]	Estimate [*SE*]	Estimate [*SE*]	Estimate [*SE*]	Estimate [*SE*]	Estimate [*SE*]	Estimate [*SE*]
*D*_Related_ = *D*_New/Neutral_	.82 [.02]	.70 [.03]	.76 [.02]	.63 [.03]	.74 [.03]	.73 [.03]	.67 [.03]
*D*_Unrelated_ = *D*_New/Related_	.77 [.02]	.64 [.02]	.71 [.02]	.57 [.02]	.66 [.02]	.66 [.02]	.63 [.02]
*d*_Related_	.74 [.06]	.51 [.10]	.69 [.07]	.60 [.09]	.60 [.08]	.72 [.06]	.64 [.07]
*d*_Unrelated_	.68 [.04]	.56 [.04]	.64 [.04]	.50 [.05]	.52 [.04]	.50 [.05]	.53 [.05]
*a* = *g*_Expected_	.58 [.05]	.64 [.04]		.61 [.03]		.54 [.03]	
*a* = *g*_Neutral_	.44 [.04]	.47 [.03]		.47 [.03]		.47 [.03]	
*b*	.51 [.03]	.32 [.02]		.42 [.02]		.46 [.03]	.54 [.02]
Model Goodness-of-fit	*G*^2^(5) = 4.48; *p* = .48	*G*^2^(13) = 14.84; *p* = .32		*G*^2^(13) = 8.15; *p* = .83		*G*^2^(12) = 8.64; *p* = .73	

*Note*. SE = standard error [values in parentheses].

### Executive dual-task conditions versus the single-task condition

The item detection parameters *D*, both for related and unrelated trials, were significantly lower in the executive dual-task conditions than in the single-task condition, *G*^2^(1), ranging from 6.51 to 44.85, all *ps* ≤ .01. For related trials, the context memory parameter d was significantly lower in the inhibition dual-task condition compared with the single-task condition, *G*^2^(1) = 4.47, *p* = .03. However, the differences between the single-task condition and the updating dual-task and the shifting dual-task conditions were not significant, *G*^2^(1) = 1.88, *ns*; *G*^2^(1) = 0.05, *ns*; respectively. For unrelated trials, the context memory parameters were significantly lower in all executive dual-task conditions than in the single-task condition, *G*^2^(1) = 4.31, *p* = .04; *G*^2^(1) = 7.93, *p* = .005; *G*^2^(1) = 8.80, *p* = .003; for single-task versus inhibition dual-task, updating dual-task, and shifting dual-task conditions, respectively.

### Executive dual-task conditions versus their respective control dual-task conditions

The *inhibition* task concurrently performed with the memory task significantly decreased item detection for unrelated trials, *G*^2^(1) = 8.00, *p* = .005, compared with the control dual task condition. In the case of related trials, item detection did not differ significantly between these two conditions, *G*^2^(1) = 2.69, *p* = .10. Moreover, in comparison with the control dual task, the inhibition task significantly decreased context memory for related trials, *G*^2^(1) = 4.28, *p* = .04, but not for unrelated trials, *G*^2^(1) = 2.03, *ns*.

The *updating* task significantly decreased item detection with no significant effect on context memory. Item detection was lower in the updating dual-task condition than in the corresponding control dual-task condition, both for related and unrelated trials, *G*^2^(1) = 7.62, *p* = .006, and *G*^2^(1) = 7.48, *p* = .006, respectively. Context memory parameters were on a very similar level in both conditions, both for related and unrelated trials, *G*^2^(1) = 0.001, *ns*; and *G*^2^(1) = 0.10, *ns*, respectively.

Performance in the *shifting* dual-task condition showed no significant differences in comparison with the corresponding control dual-task condition. No significant difference was observed in item detection or context memory, both for related and unrelated trials, *G*^2^(1), ranging from 0.20 to 2.15, all *ps* > .10.

#### Response bias

Guessing parameter *a*_Expected_ = *g*_Expected_, which refers to the tendency to guess that a word whose meaning is related to a specific colour was printed in this colour font at study, was higher than the neutral value of .50. This difference was significant in the inhibition/control dual-task condition, *G*^2^ = 14.93, *p* < .001, and in the updating/control dual-task condition, *G*^2^ = 11.90, *p* < .001, but was marginally non-significant in the single-task condition, *G*^2^ = 3.44, *p* = .06, and it was not significant in the shifting/control dual-task condition, *G*^2^(1) = 2.05, *ns*. Guessing parameter *a*_Neutral_ = *g*_Neutral_ that refers to the preference of one colour over the other study colour for words whose meaning is not related to any study colour, did not differ from the neutral value of .50, *G*^2^(1), ranging from 0.83 to 1.88, all *ps* > .10.

## Discussion

Although previous work (Nieznaski, 2013, Experiment 2) showed that a complex executive task (the RNG task) produced interference in context memory, the disturbance of specific executive functions underlying this effect could not be identified. In the current research, we selected concurrent tasks restricted to one of the three basic functions outlined by Miyake et al. (2000). The main finding of interest in the experiment was a decrease in context memory observed in the inhibition task condition for related trials. A concurrently performed task requiring the inhibition of a prepotent response disrupted item-context binding more than a similar concurrent task that required no inhibition. It seems that participants were not able to use their prior knowledge concerning the item-context association to enhance the binding of information during the study episode. As a result, in related trials they performed as poorly as in unrelated trials. Other executive tasks did not disturb context memory more than their corresponding control conditions. Alternatively, it may be argued, that the reported difference in context memory is not due to inhibition-based task interference but is solely due to the level of concurrent task difficulty. Although the concurrent tasks in dual-task experimental and their corresponding control conditions were very similar in material and response type, it is possible that the inhibition of a prepotent response makes the task especially difficult and, therefore, resource-consuming. Such a possibility cannot be definitely ruled out, but it does seem to be unlikely. First, if the inhibition task had been just a more difficult task than its control task, it would have impaired performance on unrelated trials more than on related trials. Second, the context memory parameter d was on a similar level for related trials in the inhibition dual-task condition (.51) as it was for unrelated trials in the updating or shifting dual-task conditions (.50), but it was lower than for related trials in the updating (.60) and shifting (.72) dual-task conditions. It seems that solely in the case of the inhibition dual-task condition the performance on related trials was on a similar level as on unrelated trials, whereas for the other dual-task conditions there was an advantage for related over unrelated trials. Third, if the inhibition dual task had been solely a more resource-consuming task than all other tasks, it would have impaired not only context memory but also item memory. However, in comparison with the control condition, parameter D was not significantly lower in the inhibition dual-task condition for related trials, but it was significantly lower in the case of unrelated trials.

Our results showed that concurrent updating and shifting tasks did not disturb context memory more than their control tasks that required no updating and no shifting, respectively. These results do not prove, however, that updating or shifting are not engaged in context encoding at all. It is possible that the particular tasks used in the present experiment did not engage specific resources sufficiently strongly to elicit an effect on performance. Caution in drawing conclusions should be especially exercised in the case of the shifting dual task because it did not influence both context and item memory in comparison with its control dual task. In the case of the updating dual task, although it had no effect on context memory, it significantly disrupted item memory in comparison with its control dual task. It is possible that the shifting dual task used in the experiment did not engage the shifting process sufficiently enough to influence performance, and a more difficult (and more specific) task could elicit a decrease in context memory in comparison with the corresponding control dual task. Alternatively, it may be supposed that the shifting resource is not required by the episodic memory task. In the case of updating, the effect observed for item memory suggests that the task sufficiently engaged updating processes to show the difference with its control dual task. However, the influence of the updating task was insufficient to show context memory decline, or it could be that updating is not important for context memory. In future research, other executive tasks have to be used to confirm the importance of the inhibition process and to verify the lack of importance of shifting and updating processes for context memory, as preliminarily suggested by the current research.

The single-task condition resulted in better item memory performance in comparison with all the executive dual-task conditions. However, in the case of context memory, the impact of inhibition, updating, and shifting tasks was significant only for unrelated trials. In the case of related trials, only the inhibition task significantly disturbed context memory. The more salient influence of cognitive load on unrelated trials than related trials confirms earlier results with the generation task as a resource-limiting factor (Nieznaski, 2013).

Summing up, the results of the present experiment suggest that item-context binding and the inhibition of the prepotent response may require the same executive resource because the parallel performance of these tasks causes a significant decrease in context memory. Moreover, comparisons between the single-task condition and all executive dual-task conditions suggest that a general cognitive resource is required to successfully perform a context memory task. When item-context binding was difficult (on unrelated trials), performance was more dependent on the available resources than when binding was easy (on related trials). The results showed that the inhibition task has a specific impact on item-context binding, which is apparent on related trials.

If we assume, following Baddeley (2000), that the episodic buffer plays an important role in encoding and retrieving information from LTM, the present results suggest that a disturbance of the inhibition process impairs the usage of pre-experimental associations in binding item and context information. Referring Cowan’s (1999) model to our experiment, we can assume that the features of items and their contexts, when being in the focus of attention, are more or less effectively bound and a composite trace is encoded into LTM. For related trials, item and context features are already associated with each other in the LTM. Therefore, their composite trace may be easily accessed and function as if it was held in an activated form in memory (Cowan calls this readily accessible portion of LTM a “virtual short term memory”). Our results suggest that this access to virtual short term memory may be impaired due to inhibition required by concurrent task performance. A similar interpretation may be based on Oberauer’s (2009; Oberauer & Hein, 2012) three-embedded-components model. In this model, the main function of the central component (i.e., the region of direct access, DA) is to build and maintain new bindings between representational elements. We may assume that this DA region provides bindings between words and their contexts in our experimental paradigm. Another component of WM is the activated part of LTM, representations activated in LTM may be projected into the DA region and increase the efficiency of processing, which probably occurs for related trials in the single-task condition of our experiment. However, it seems that during inhibition in the dual-task condition, the threshold is raised for information activated in the LTM and performance for related trials is not better than for unrelated trials. Finally, our results can be referred to Engle’s views of WM capacity (e.g., Engle, 2002; Engle et al., 1999). According to this approach, WM capacity is not directly about memory storage—it is about the capacity for controlled, sustained attention, particularly in the face of interference or distraction, as is the case in dual-task experiments. A greater WM capacity means a greater ability to use attention to maintain or suppress information. As pointed out by Redick, Heitz, and Engle (2007), inhibition is a controlled and resource-demanding process. Therefore, it seems that inhibitory ability and item-context binding both rely on WM capacity. It seems that the models mentioned above, explain the role of WM capacity for item-context binding quite well. However, they do not account so well for the differences between effects of the specific executive resources, we found in our experiment. Future studies should examine the issue further.

The last point that has to be discussed here is the assumption concerning the facilitating influence of prior knowledge on item-context binding in related trials. As Johnson and colleagues (Johnson, Hashtroudi, & Lindsay, 1993; Johnson & Raye, 2001) stated in their source monitoring framework, source (context) attributions can be influenced by prior knowledge, schemas, or expectations. In accordance with this prediction, in the current experiment and in earlier experiments (Nieznaski, 2013), cognitive load mostly resulted in worse context memory for unrelated trials than for related trials. However, it is not always the case that related item-context pairings are better remembered than unrelated pairings. For example, in the study by Bayen et al. (2000), mentioned earlier in the text, there was no memory advantage for expected context. Also, many other studies found equal memory for expected and unexpected contexts (e.g., Bayen & Kuhlmann, 2011; Kuhlmann, Vaterrodt, & Bayen, 2012). Moreover, in recent experiments by Küppers and Bayen (2014), worse context memory for expected than unexpected contexts has been shown. These effects were explained in accordance with the attention-elaboration hypothesis (cf. Erdfelder & Bredenkamp, 1998), which states that schema-inconsistent information attracts more attention and undergoes deeper elaboration than schema-consistent information. Hence, a very unexpected context is better encoded than an expected one. It would seem that these results from the literature are at odds with results reported here and by Nieznaski (2013). However, note that the type of context that was used here was quite different from that used in experiments confirming the attention-elaboration hypothesis. Moreover, reliance on background knowledge may depend on cognitive load and the participants’ readiness to deliberately discern the item-context relationship during encoding (e.g., Hicks & Cockman, 2003; Konopka & Benjamin, 2009). To the best of our knowledge, all the studies reporting a null or positive effect of inconsistency on context memory have used extrinsic contexts (i.e., attributes which are external to a target item) (e.g., pictures and names of a doctor or lawyer, words describing a scene—bathroom or bedroom). In our experiments, we used an intrinsic context (font colour), which refers to the inevitably processed physical attribute of an item. Many studies have shown that extrinsic and intrinsic context information are differently processed and represented in memory. For example, Mulligan (2011) and Nieznaski (2012, 2014) have demonstrated that generating an item results in an increase in memory for extrinsic context but a decrease in memory for intrinsic context (see Boywitt & Meiser, 2012; Ecker, Maybery, & Zimmer, 2013; Ecker, Zimmer, & Groh-Bordin, 2007; Geiselman & Bjork, 1980; Godden & Baddeley, 1980; Troyer & Craik, 2000; for studies showing differential consequences of processing intrinsic vs. extrinsic context). The explanation why expectancy effects seem to be different for extrinsic versus intrinsic contexts needs future experimental investigation.

## Footnotes

1 As noted by one of the reviewers, participants who completed the inhibition task during the second session (i.e., after completing the control session) may have experienced more difficulty in inhibiting the prepotent response than those who completed the inhibition task in the first session. In order to check if this influenced our results, we compared the context memory performance of the participants who completed the inhibition task during the first session with the performance of those who completed it during the second session. Surprisingly, context memory parameters d were slightly numerically higher when the inhibition task was completed during the second session than during the first session, which suggests that interference from the inhibition task was not stronger during the second session than during the first session; *d*_Related_ = .26 versus .37 (*G*^2^(1) = .38, *ns*) and *d*_Unrelated_ = .56 versus .65 (*G*^2^(1) = 1.53, *ns*), for the first versus second session performance, respectively.

2 This operational assumption is borrowed from the two-high-threshold model of recognition memory. Snodgrass and Corwin (1988, p. 38) argued that this equality assumption is warranted by the *mirror effect* in recognition—as hit rates increase across various manipulations, the corresponding false alarm rates decrease in an inverse fashion.

## Appendix A

**Table A1.** Response Frequencies Obtained in the Experiment

Condition	Single task			Inhibition dual task			Control to Inhibition		
Trial type	Correct	Incorrect	New	Correct	Incorrect	New	Correct	Incorrect	New
Related Colour	393	63	48	275	68	89	311	48	73
Opposite Colour	336	108	60	206	111	115	265	89	78
Colour 1 / Neutral colour related	173	53	26	129	43	44	127	47	42
Colour 2 / Neutral colour related	188	36	28	121	39	56	134	38	44
	Expected	Unexpected	“New”	Expected	Unexpected	“New”	Expected	Unexpected	“New”
New / Colour 1 or 2 related	35	27	442	36	13	383	27	19	386
	“Colour 1”	“Colour 2”	“New”	“Colour 1”	“Colour 2”	“New”	“Colour 1”	“Colour 2”	“New”
New / Neutral colour related	13	8	231	6	13	197	8	6	202

**Table A1. cont.**

Updating dual task			Control to Updating			Shifting dual task			Control to Shifting		
Correct	Incorrect	New	Correct	Incorrect	New	Correct	Incorrect	New	Correct	Incorrect	New
271	70	91	296	69	67	302	64	66	281	62	69
212	115	105	231	122	79	236	111	85	251	115	66
103	59	54	123	45	48	121	58	37	119	60	37
115	49	52	117	52	47	132	49	35	126	49	41
Expected	Unexpected	“New”	Expected	Unexpected	“New”	Expected	Unexpected	“New”	Expected	Unexpected	“New”
51	28	353	38	22	372	42	28	362	47	43	342
“Colour 1”	“Colour 2”	“New”	“Colour 1”	“Colour 2”	“New”	“Colour 1”	“Colour 2”	“New”	“Colour 1”	“Colour 2”	“New”
14	21	181	9	13	194	10	14	192	21	14	181
